# Chemical Composition, Antibacterial Activity, and Synergistic Effects with Conventional Antibiotics and Nitric Oxide Production Inhibitory Activity of Essential Oil from *Geophila repens* (L.) I.M. Johnst

**DOI:** 10.3390/molecules22091561

**Published:** 2017-09-17

**Authors:** Huijuanzi Rao, Pengxiang Lai, Yang Gao

**Affiliations:** Marine College, Shandong University, Weihai 264209, China; sd15juanzi@163.com (H.R.); wh_xiang6@163.com (P.L.)

**Keywords:** *Geophila repens* (L.) I.M. Johnst, essential oil, GC-MS, antibacterial, synergistic, Checkerboard method, nitric oxide

## Abstract

*Geophila repens* (L.) I.M. Johnst, a perennial herb, belongs to the *Rubiaceae* family. In this study, we identified the chemical composition of the *Geophila repens* essential oil (GR-EO) for the first time. Totally, seventy-seven compounds were identified according to GC and GC-MS, which represent 98.0% of the oil. And the major components of GR-EO were β-caryophyllene (23.3%), β-elemene (8.0%), farnesyl butanoate (7.4%), myrcene (3.5%), and *trans*-nerolidol (3.3%). Then we evaluated the antibacterial activities of GR-EO and the synergistic effects of GR-EO in combination with commercial antibiotics using the microdilution and Checkerboard method. The results demonstrated that GR-EO possessed an excellent broad spectrum antibacterial activity, especially against *Pseudomonas aeruginosa* and *Bacillus subtilis*. It also showed that the combined application of GR-EO with antibiotics led to synergistic effects in most cases. And the most prominent synergistic effect was noticed when GR-EO was in combination with Streptomycin and tested against *Escherichia coli* (fractional inhibitory concentration indices (FICI) of 0.13). Additionally, the results of a Griess assay revealed that GR-EO exhibited a potent inhibitory effect on NO production in lipopolysaccharide (LPS)-activated RAW 264.7 (murine macrophage) cells. In conclusion, the combination of GR-EO and the commercial antibiotics has significant potential for the development of new antimicrobial treatment and reduction of drug resistance.

## 1. Introduction

At present, antibiotic resistance is one of the greatest threats to public health, so it is clear that searching for new antibacterial agents is absolutely necessary. Antibiotic resistance can be intrinsic or acquired and can be transmitted within the same or different species of bacteria. The mechanisms can be divided into different categories: (i) modification of the active site of the target resulting in a reduction in the efficiency of binding of the antibiotic; (ii) direct destruction or modification of the antibiotic by enzymes produced by the bacterium; (iii) efflux or removal of antibiotic from the cell cause a reduced amount of antibiotic; or (iv) production of an alternative target that is resistant to inhibition by the antibiotic (metabolic by-pass) [1,2,3].

Exploring the natural products from plants could become an interesting alternative therapy [3,4,5]. Some studies demonstrated that the natural products form plants may modulate the action of antibiotics by increasing or decreasing their activities [2,6,7]. Therefore, to find antimicrobial plant products which can improve the antibacterial effect of commonly used antibiotics may be an effective way to overcome bacterial resistance [8,9,10]. Essential oils are a very interesting group of secondary metabolites that are potentially useful sources of antimicrobial compounds, and they are composed of many molecules so that bacteria cannot resist in mutant [11]. Many studies have demonstrated that essential oils of plants exhibit not only interesting antimicrobial properties, but possess the ability to enhance the activity of antibiotic [4,8,11,12,13]. The combination therapy, i.e., essential oils and antibiotics, tends to employ several mechanisms of action to combat pathogens, such as sequentially inhibiting a common biochemical pathway, inhibiting protective enzymes and using cell wall active agents to enhance the uptake of antibiotics [14]. Consequently, they can be a powerful tool to reduce the bacterial resistance [12,15].

The *Rubiaceae* family contains about 13,500 species and 611 genera. It consists of terrestrial trees, shrubs, lianas, and herbs. *Rubiaceae* has a cosmopolitan distribution. However, the largest species diversity is concentrated in the tropics and subtropics [16]. *Geophila* is a small genus of the *Rubiaceae* family, containing about 30 species. It is found primarily in Asia-Tropical and Africa.

*Geophila repens* (L.) I.M. Johnst belongs to the *Rubiaceae* family and is widely distributed in the tropics and subtropics of Africa, Asia, through the Pacific to the Americas and Caribbean. It is a creeping, evergreen, perennial herb growing up to 10 cm tall and often forming dense close colonies in shaded places. The leaves of *Geophila repens* are sometimes chewed to remedy coughs. And a decoction of the boiled plant is also used as a treatment for coughs. The fruits of *Geophila repens* are considered to be an effective antifungal agent [17].

In the present study, we extracted the *Geophila repens* essential oil (GR-EO) by hydrodistillation, and identified the chemical composition of GR-EO using GC and GC-MS for the first time. Then we demonstrated the in vitro antibacterial properties of GR-EO against four bacterial strains by disc agar diffusion and broth microdilution methods, and studied the synergistic effect of the combination of GR-EO with standard antibiotics using Checkerboard method. In addition, we evaluated the inhibitory effects of GR-EO on NO production in lipopolysaccharide (LPS)-activated murine macrophage RAW 264.7 cells.

## 2. Results

### 2.1. Chemical Composition

The components of the essential oil with their retention indices (RI) from the *Geophila repens* are listed in Table 1. The analysis of the oil by Agilent 6890 gas chromatograph (Agilent, Santa Clara, CA, USA) equipped with flame ionization detector (FID, Agilent) and GC-MS showed the presence of 77 constituents that were identified, which accounts for almost 98.0% of the oil. Sesquiterpene hydrocarbons were predominant (45.2%), followed by oxygenated sesquiterpenes (25.9%), and monoterpene hydrocarbons (7.3%), diterpenes (4.7%), and oxygenated monoterpenes (3.0%). The major chemical constituents were found to be β-caryophyllene (23.3%), β-elemene (8.0%), farnesyl butanoate (7.4%), myrcene (3.5%), *trans*-nerolidol (3.3%), and α-caryophyllene (3.3%).

### 2.2. Antibacterial Activity

GR-EO was tested against four pathogenic bacteria, including two gram-positive bacteria (*S. aureus* and *B. subtilis*) and two gram-negative bacteria (*E. coli* and *P. aeruginosa*). The antibacterial activities of GR-EO were qualitatively assessed by the presence or absence of inhibition zone diameters (DIZs). The results are presented in Table 2. GR-EO exhibited obvious antibacterial activities against most tested bacteria strains with DIZs of (24.1 ± 0.6), (23.8 ± 0.7), and (15.8 ± 0.5) mm for *P. aeruginosa*, *B. subtilis,* and *S. aureus*, respectively. However, this oil showed low activity (DIZ < 7.5 mm) towards *E. coli*.

More accurate data on the antibacterial activity were obtained through the MIC and MBC (Minimal bactericidal concentration) values of the oil. From the data, it is evident that GR-EO displayed a broad spectrum antibacterial activity against the tested bacterial strains. As shown in Table 2, the most susceptible strains were *P. aeruginosa* (MIC = 0.049 mg/mL, MBC = 0.049 mg/mL) and *B. subtilis* (MIC = 0.049 mg/mL, MBC = 0.049 mg/mL), followed by *S. aureus* (MIC = 0.250 mg/mL, MBC = 0.500 mg/mL). However, it showed a weak activity against *E. coli* (MIC = 1.563 mg/mL, MBC = 3.125 mg/mL).

### 2.3. Evaluation of Synergistic Effects

The evaluations of synergistic effects of GR-EO in combination with four selected conventional antibiotics were determined using the Checkerboard method. The interaction data in the form of the fractional inhibitory concentration indices (FICIs) are listed in Table 3, Table 4, Table 5 and Table 6, respectively. The meaning of FICI as synergistic effect (≤0.5), additional effect (0.5–1), indifference effect (1–4), and antagonism (≥4), were described previously [19].

#### 2.3.1. Evaluation of Synergistic Effects with Chloramphenicol

Synergistic effects of Chloramphenicol combined with GR-EO according to the calculated FICIs are presented in Table 3. Combination of the GR-EO with Chloramphenicol showed a synergistic effect against *B. subtilis* (FICI of 0.38), *P. aeruginosa* (FICI of 0.38), and *E. coli* (FICI of 0.5). While an indifference effect was observed in *S. aureus* (FICI of 1.5)*.*

#### 2.3.2. Evaluation of Synergistic Effects with Streptomycin

According to the obtained results presented in Table 4, a significant synergistic effects was observed in *E. coli* (FICI of 0.13), *P. aeruginosa* (FICI of 0.38) and *S. aureus* (FICI of 0.38) when there was a combination of GR-EO with Streptomycin, while an additive effect was seen in *B. subtilis* (FICI of 0.75).

#### 2.3.3. Evaluation of Synergistic Effects with Penicillin

Combination of GR-EO and Penicillin displayed synergistic activity against both *P. aeruginosa* (FICI of 0.25) and *E. coli* (FICI of 0.5) according to Table 5. The combination against strains of *S. aureus* and *B. subtilis* were not performed because these strains were sensitive when antibiotic was administered alone.

#### 2.3.4. Evaluation of Synergistic Effects with Ampicillin

As is presented in Table 6, combination of GR-EO and Ampicillin led to a synergistic effect against *P. aeruginosa* (FICI of 0.38) and an additive effect against *E. coli* (FICI of 1). The combination against strains of *S. aureus* and *B. subtilis* were not performed because these strains were sensitive when antibiotic was administered alone.

### 2.4. Inhibitory Effect on the NO Production of RAW 264.7

**Figure 1:**
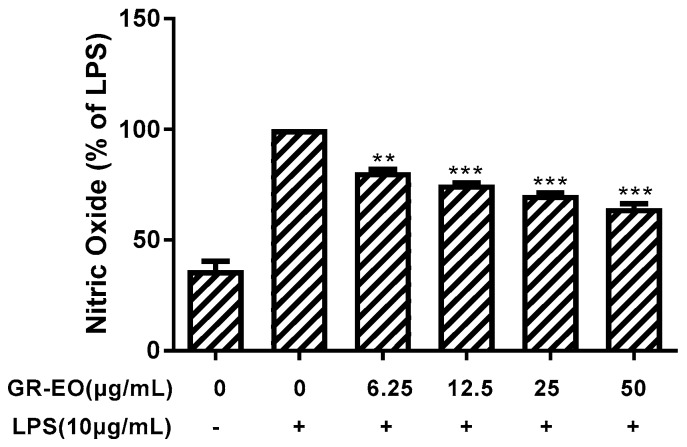
NO production with GR-EO in RAW 264.7 Cells. The cells were incubated with LPS (10 μg/mL) only and with LPS plus the indicated concentrations of GR-EO for 24 h. Results were expressed as a percentage of NO production by the LPS-treated cells (LPS group). All values were performed in triplicate and expressed as mean ± SD. ** *p* ≤ 0.01 and *** *p* ≤ 0.001 compared with the LPS only group.

We determined the inhibitory effect of GR-EO on the NO production of RAW 264.7 macrophages using Griess reagent system. As shown in Figure 1, the essential oil exhibited significant inhibitory effect on the LPS-stimulated cells in a dose-dependent manner. It inhibited NO release by 19.35%, 24.94%, 29.66%, and 35.51% compared with LPS treated cells at the concentration of 6.25, 12.5, 25, and 50 μg/mL, respectively. In the MTT assay, GR-EO was noncytotoxic by themselves at concentrations up to 50 μg/mL in RAW 264.7 cells (data was not shown), which suggested that the inhibitory activity against NO production in LPS-activated RAW 264.7 cells was not induced by the cytotoxicity of the tested GR-EO.

## 3. Discussion

In the present study, the major constituents in the GR-EO were found to be β-caryophyllene (23.3%), β-elemene (8.0%), farnesyl butanoate (7.4%), myrcene (3.5%), *trans*-nerolidol (3.3%), and α-caryophyllene (3.3%). It is noteworthy that some major compounds had been reported to have various bioactivities. Such as β-caryophyllene, a natural bicyclic sesquiterpene, which is a constituent of many essential oils, especially clove oil. Previous studies have indicated that β-caryophyllene possessed a significant activity against gram-positive bacteria with MIC values from 0.032 to 0.256 mg/mL [20]. Further studies revealed that β-caryophyllene had the potential ability to suppress tumor motility, cell invasion, and tumor aggregation [20,21]. Another main component is β-elemene, which has been shown to inhibit tumor cell growth in vitro and in vivo, and has been put into clinical trials in cancer patients in China [22,23,24].

According to the results of antimicrobial activity testing, GR-EO had a potent effect on both gram-negative and gram-positive bacteria. The mechanisms of the antimicrobial action of essential oils are still not completely understood. Some studies suggested that, the amphiphilic nature of terpenoids due to the presence of hydrophobic skeleton and hydrophilic functional groups enables easy transport of these phytochemicals across biological membranes of microbial cells, thereby allowing them to reach cytosol and interact with key intracellular biomolecules [12,25]. Generally, the antibacterial properties of essential oils are closely associated with their most abundant components therein [26]. Although the previous studies revealed a significant antibacterial activity of β-caryophyllene [20], the antibacterial effect of the oil was higher than that of single constituent of β-caryophyllene. Some studies have demonstrated that different terpenoid components of essential oils can interact to either reduce or increase antimicrobial efficacy [27]. However, in many cases the whole oils usually have higher antibacterial capacity than the mixtures of their major components, suggesting that the minor components are critical to the synergistic activity [28,29,30]. Therefore it was speculated that a synergistic effect between the major and minor components of GR-EO contributed to the antibacterial activity.

Furthermore, the synergistic effects of GR-EO in combination with four selected conventional antibiotics were evaluated against *B. subtilis*, *S. aureus*, *P. aeruginosa,* and *E. coli*. The present study revealed the synergistic effect of GR-EO in combination with Chloramphenicol, Streptomycin, Penicillin, or Ampicillin against a wide range of bacterial strains. It is to be noted that remarkable synergistic action was observed against *E. coli* by the combination of GR-EO with Streptomycin (FICI of 0.13). *Escherichia coli*, a gram-negative bacterium, has been linked to numerous common bacterial infections, including bacteremia, cholecystitis, cholangitis, urinary tract infection (UTI), and other clinical infections such as neonatal meningitis and pneumonia [31]. Therefore, the combination therapy involving Streptomycin and GR-EO can represent a novel approach in the management of several infections. Besides, the combination of GR-EO with Chloramphenicol or Penicillin also possessed synergistic effects against *E. coli* with the FICI values of 0.5.

From the results obtained, what is more noteworthy is that the combinations of GR-EO plus all tested antibiotics have conspicuous synergistic effects when tested against *P. aeruginosa* (FICI range of 0.25 to 0.38). *Pseudomonas aeruginosa*, a gram-negative bacterium, is one of the leading causes of nosocomial infections. Severe infections, such as pneumonia or bacteremia, are associated with high mortality rates and are often difficult to treat, as the repertoire of useful anti-pseudomonal agents is limited; moreover, *P. aeruginosa* has high intrinsic resistance to antibiotic and exhibits remarkable ability to acquire resistance to these agents during treatment [32]. The ability of antibiotic resistance of *P. aeruginosa* is significantly due to low permeability of the cell wall, which restricts the uptake of antibiotics, and the genetic capacity to express a wide repertoire of resistance mechanisms, like efflux pumps and enzymes, which modify or degrade antibiotics and drug targets [33]. Therefore, combination therapy involving GR-EO tends to open new avenues and a viable treatment option.

Additionally, synergy was also noted for another two interactions, namely GR-EO in combination with Chloramphenicol against *B. subtilis* (FICI of 0.38), and with Streptomycin against *S. aureus* (FICI of 0.38). *Staphylococcus aureus* is a major human pathogen that causes a wide range of clinical infections. It is a leading cause of bacteremia and infective endocarditis as well as osteoarticular, skin and soft tissue, pleuropulmonary, and device-related infections [34]. Therefore, treatment options of *S. aureus* infections could include combined use of GR-EO and Streptomycin.

There are fewer reports on the mode of action of the essential oils in combination with antibiotics. And some mechanisms of antimicrobial interaction that produce synergism are generally accepted, such as the sequential inhibition of a common biochemical pathway, inhibition of protective enzymes, and use of cell wall active agents to enhance the uptake of other antimicrobials [14].

This study indicates that the combination of GR-EO and the commercial antibiotics has significant potential for the development of new antimicrobial treatment and reduction of drug resistance, which will permit to find the treatment of several infections caused by microorganisms.

Additionally, we also evaluated the inhibitory effects of GR-EO on NO production in lipopolysaccharide (LPS)-activated murine macrophage RAW 264.7 cells. LPS is a major component of the outer membrane of gram-negative bacteria, and it has been recognized as important in determining both the virulence of these organisms and the symptomatology that accompanies gram-negative bacteria infection [35]. When bacterial cells are lysed by the immune system, fragments of membrane containing LPS are released into the circulation. After exposure to LPS from gram-negative bacteria, inducible NOS (iNOS) can be induced in various cells, such as macrophages, kupffer cells, and hepatocytes, to trigger cytotoxicity, tissue damage, inflammation sepsis, and stroke, and can cause fever, diarrhea, and possible fatal endotoxic shock. Therefore, LPS is in large part responsible for the dramatic clinical manifestations of infections with pathogenic gram-negative bacteria [36,37].

The evaluation of NO production showed that GR-EO presented a significant inhibitory effect. It could be attributed to the presence of significant amounts of β-caryophyllene and *trans*-nerolidol, which have been reported to possess excellent inhibitory effect on the NO production by LPS-stimulated macrophages [37,38]. Additionally, in vitro studies suggest that β-caryophyllene is a dietary cannabinoid with important anti-inflammatory effects through the inhibition of the cannabinoid type-2 receptor. Meanwhile, in vivo, peroral β-caryophyllene strongly reduces inflammation in a mouse model of colitis [39].

## 4. Materials and Methods

### 4.1. Plant Samples

*Geophila repens* was collected in July 2016 from Guangxi Province of China, and was identified by Associate Prof. Hong Zhao of Shandong University. A voucher specimen (No. 10807) was deposited at the Laboratory of Botany of Marine College, Shandong University.

### 4.2. Isolation of Essential Oil

The fresh plant material (600 g) was hydrodistilled for five hours using a Clevenger apparatus to extract GR-EO (0.39 g, 0.065% *w*/*w*). The oil was stored at 4 °C in dark vials until analysis.

### 4.3. Gas Chromatography Analysis

Analysis of GR-EO was performed by gas chromatograph (GC) using an Agilent 6890 (Agilent, Santa Clara, CA, USA) equipped with flame ionization detector (FID, Agilent). GC-FID analysis of the oil was carried out on HP-5 MS capillary column (30 m × 0.25 mm, 0.25 μm film thickness) under the following conditions: Program the oven temperature stay at 60 °C for 1 min, then heat to 280 °C at a rate of 6 °C/min, hold at 280 °C for 2 min. The carrier gas used was helium at a constant flow rate of 1.2 mL/min, while the detector and injector temperatures were 250 °C and 240 °C, respectively.

### 4.4. Gas Chromatography-Mass Spectrometry Analysis

The GC-MS analysis was carried out on Hewlett Packard 6890 gas chromatograph (Agilent) attached to a HP-5MS fused silica column, interfaced with a Hewlett Packard 5975C mass selective detector operated by HP Enhanced ChemStation software (Agilent). The analytical conditions for GC were the same as those mentioned for GC-FID. The injection volume was 0.2 μL of 1% solution prepared in n-hexane with split ratio 1:50. Mass spectra were acquired in EI mode at 70 eV. The mass range was from *m*/*z* 50 to 550.

### 4.5. Identification of Components

Identification of the constituents was performed on the basis of their retention time, retention indices (relative to C_7_–C_30_ n-alkanes, under the same experimental conditions), and computer matching with Nist MS Search 2.2 Mass Spectral Database for GC-MS as well as comparisons of their mass spectra with those of authentic samples or with data already available in the literature. The result of the analysis is shown in Table 1.

### 4.6. Antibacterial Activity

#### 4.6.1. Microbial Strains and Culture

The antibacterial activity of the investigated essential oil was tested against four bacteria strains. The following microorganisms were used in this work: two gram-positive (*Bacillus subtilis* ATCC 6633; *Staphylococcus aureus* ATCC 6538) and two gram-negative (*Pseudomonas aeruginosa* ATCC 27853; *Escherichia coli* ATCC 25922) bacteria. All strains were cultured at 37 °C on Mueller-Hinton medium.

#### 4.6.2. Preparation of the Bacterial Inoculums

Each strain was grown in liquid nutrient broth at 37 °C for 18 h before being used for antimicrobial tests. The microbial load was adjusted to 10^8^ Colony-Forming Units (CFU)/mL using a 0.5 McFarland turbidity standard, after that, the suspension was diluted to obtain an inoculum of 10^6^ CFU∙mL^−1^.

#### 4.6.3. Diameter of Inhibition Zone (DIZ) Determination

Antibacterial activities were assessed by the disc agar diffusion method recommended by CLSI (Clinical and Laboratory Standards Institute) (2012) [40]. Briefly, each strain was incubated overnight in MHB and then adjusted to 1 × 10^6^ CFU/mL. Then 100 μL of bacterial suspension was spread on the MHA plates, on which filter paper discs (6 mm in diameter) individually saturated with essential oil (10 μL, 10 mg/mL) were placed. The plates were incubated at 37 °C for 24 h. The DIZ values were measured with a caliper in millimeter. Chloramphenicol (10 μg/disc) was used as the control. All the tests were repeated in triplicate.

#### 4.6.4. Determination of Minimal Inhibitory Concentration (MIC)

The MIC values were performed in 96 well-microplates using the microdilution assay according to the literature previously described by Ellof (1998) with slight modifications [41]. The essential oil was dissolved in dimethyl sulfoxide (DMSO), and diluted with MHB to a final concentration with DMSO <4%, then transferred into each well (100 μL per well). Chloramphenicol was used as the reference antibiotic control. The inoculum was added to all wells (100 μL per well). And bacterial suspension containing DMSO (4%) without EO was used as control. The plates were incubated at 36 °C for 12–16 h. Antimicrobial activity was detected by adding 20 μL of 1% TTC (2,3,5-triphenyl tetrazolium chloride) aqueous solution. MIC was defined as the lowest concentration of the essential oils that inhibited visible growth, as indicated by TTC staining [42]. Experiments were carried out in triplicates to minimize experimental error.

#### 4.6.5. Determination of Minimum Bactericidal Concentration (MBC)

For the determination of the MBC, a sample of 100 μL from each well (without any color alteration) was subcultured on the MHA plates and incubated at 36 °C for 18–24 h (overnight). The MBC is defined as the lowest concentration without any bacterial growth.

### 4.7. Evaluation of Synergistic Effects

The synergistic effect of triterpenoids in combination with antibiotics was determined using the Checkerboard dilution method [43]. According to the MIC values of GR-EO and antibiotics, GR-EO and antibiotics were diluted by MHB to various concentrations ranging from 1/64 to four fold of their MICs, and transferred to the 96 well-microplates (50 μL per well). The antibiotic was serially diluted along the vertical axis while the essential oil was serially diluted along the horizontal axis so that each well represents a unique concentration of the combination being tested. Then 100 μL of previously prepared inoculum was then added to each well. The plates were incubated at 36 °C for 12–16 h. After the incubation period, 20 μL of 1% TTC was added to the wells and left to incubate for an additional 30 min. The wells at the growth–no growth interface were determined and the fractional inhibitory concentration index (FICI) of the combination was calculated according to the equation below:

(1) $$\mathrm{FICI}=\frac{\mathrm{MIC}\;\mathrm{of}\;\mathrm{EO}\;\mathrm{in}\;\mathrm{combination}}{\mathrm{MIC}\;\mathrm{of}\;\mathrm{EO}\;\mathrm{alone}}+\frac{\mathrm{MIC}\;\mathrm{of}\;\mathrm{antibiotic}\;\mathrm{in}\;\mathrm{combination}}{\mathrm{MIC}\;\mathrm{of}\;\mathrm{antibiotic}\;\mathrm{alone}}.$$

### 4.8. Inhibitory Effect on the NO Production of RAW 264.7

#### 4.8.1. Cell Culture

The murine macrophage cell line RAW 264.7 was cultured in DMEM (Dulbecco’s modified eagle medium) supplemented with 10% FBS, 100 U/mL of penicillin and 100 μg/mL of streptomycin. All cells were grown at 37 °C in 5% CO_2_ and humidified air atmosphere.

#### 4.8.2. MTT Assay

The cytotoxicity of GR-EO was evaluated by MTT assays. GR-EO was solubilized in DMSO, and afterwards diluted with culture medium for use. RAW 264.7 cells (5 × 10^3^ cells per well) with 200 μL of culture medium were seeded in 96-well plates and grown for 24 hours to allow cell attachment. After that, the cells were treated with or without the dilutions of the oil for 24 h. Then, 20 μL MTT solutions (5 mg/mL in PBS) were added to each well for another 4 h and the resulting crystals were dissolved in dimethyl sulfoxide (DMSO). The optical density was read at 570 nm. Cytotoxicity was calculated from the plotted results using untreated cells at 100%. The percentages of cell growth were calculated as follows:

Cell growth (%) = [A (sample) / A (control)] × 100%.(2)

#### 4.8.3. Nitrite Assay with a Griess Reagent

To investigate the anti-inflammatory activity of GR-EO, NO production was assessed according to the Griess reaction [44]. Each tested sample was dissolved in DMSO, and diluted with fresh FBS-free DMEM media to a final concentration with DMSO ≤ 0.1%. The RAW 264.7 macrophages were seeded in 96-well plates (5 × 10^4^ cells/mL) and co-incubated with samples and LPS (10 μg∙mL^−1^). After incubation at 37 °C for 24 h, each 50 μL of the culture supernatant was mixed with 50 μL of Griess reagent I and 50 μL of Griess reagent II at room temperature. Absorbance was measured at 540 nm using a microtiter plate reader. A series of known concentrations of sodium nitrite (NaNO_2_) was used as the standard curve.

### 4.9. Statistical Analysis

All of the tests were conducted in triplicate and the experimental data were expressed as means ± SD. Statistical analysis was performed by Student’s *t*-test. Differences were considered significant at *p* ≤ 0.05. GraphPad, Prism version 7.0 was used for statistical analysis.

## 5. Conclusions

The current study identified the chemical components of GR-EO by GC and GC-MS, evaluated the antibacterial activity of GR-EO alone and the synergistic effects of the combinations between GR-EO and antibiotics, and assessed the NO production inhibitory effect. The results obtained revealed that GR-EO exhibits potent inhibitory effect on LPS-induced NO production in RAW 264.7 macrophages, and possesses significant antibacterial effect, especially against *P. aeruginosa* and *B. subtilis*. Additionally, synergistic effects were observed against tested bacteria when GR-EO was combined with conventional antibiotics, which could lead to new options for the treatment of infectious diseases caused by bacteria and emerging drug resistance. Moreover, no antagonistic effect was observed in this study further augments the potential for the use of GR-EO in combination with antibiotics.

## Figures and Tables

**Table 1:** Chemical composition of *Geophila repens* essential oil (GR-EO).

Peak No.	Compound ^a^	RI ^b^	RI ^c^	Peak Area %	Identification ^d^
1	β-Pinene	961	961	0.4	MS, RI
2	Myrcene	992	991	3.5	MS, RI
3	3-Carene	1033	1031	2.0	MS, RI
4	γ-Terpinene	1061	1061	0.1	MS, RI
5	Linalol	1100	1100	1.8	MS, RI
6	(4*E*,6*Z*)-*allo*-Ocimene	1131	1131	0.3	MS, RI
7	Chrysanthenol	1145	1144	0.1	MS, RI
8	Camphor	1155	1152	0.2	MS, RI
9	4-Terpineol	1185	1187	0.6	MS, RI
10	Terpineol	1197	1196	0.3	MS, RI
11	Decanal	1205	1205	3.0	MS, RI
12	(*E*)-2-Decenal	1278	1276	0.3	MS, RI
13	α-Tridecene	1287	1287	0.4	MS, RI
14	2-Undecanone	1293	1293	0.9	MS, RI
15	Undecanal	1306	1306	0.3	MS, RI
16	α-Longipinene	1337	1342	0.1	MS, RI
17	δ-Elemene	1347	1346	1.3	MS, RI
18	α-Cubebene	1359	1360	0.5	MS, RI
19	Eugenol	1369	1366	0.1	MS, RI
20	β-Patchoulene	1378	1377	0.1	MS, RI
21	α-Ylangene	1383	1377	0.3	MS, RI
22	Copaene	1389	1390	0.8	MS, RI
23	β-Elemene	1404	1403	8.0	MS, RI
24	Dodecanal	1411	1411	2.0	MS, RI
25	Isocaryophyllene	1424	1425	0.3	MS, RI
26	Caryophyllene	1441	1444	23.3	MS, RI
27	(*Z*)-β-Farnesene	1458	1457	0.6	MS, RI
28	γ-Himachalene	1467	1468	0.4	MS, RI
29	α-Caryophyllene	1472	1474	3.3	MS, RI
30	γ-Muurolene	1480	1481	0.4	MS, RI
31	Eremophilene	1491	1486	1.1	MS, RI
32	Pentadecane	1500	1500	1.8	MS, RI
33	α-Selinene	1506	1504	1.3	MS, RI
34	β-Eudesmene	1514	1509	2.5	MS, RI
35	δ-Cadinene	1531	1531	0.4	MS, RI
36	α-Cadinene	1536	1536	1.2	MS, RI
37	γ-Cadinene	1549	1552	0.5	MS, RI
38	Caryophyllene oxide	1553	1552	0.3	MS, RI
39	β-Calacorene	1561	1561	0.4	MS, RI
40	(*E*)-Nerolidol	1569	1569	3.3	MS, RI
41	Denderalasin	1581	1581	0.9	MS, RI
42	Isoaromadendrene epoxide	1594	1594	0.5	MS, RI
43	Cedrol	1601	1599	1.4	MS, RI
44	Caryophyllene oxide	1607	1607	1.8	MS, RI
45	Cubenol	1622	1623	0.3	MS, RI
46	Neointermedeol	1631	1631	0.6	MS, RI
47	δ-Cadinol	1645	1645	1.4	MS, RI
48	τ-Cadinol	1662	1660	1.6	MS, RI
49	Globulol	1675	1675	1.4	MS, RI
50	α-Asarone	1681	1678	0.6	MS, RI
51	Bisabolol	1694	1693	0.8	MS, RI
52	Isolongifolol	1699	1695	1.0	MS, RI
53	(*Z*)-β-Santalol	1711	1720	1.1	MS, RI
54	Farnesol	1744	1747	0.8	MS, RI
55	(*Z*)-Lanceol	1764	1764	0.3	MS, RI
56	β-Costol	1774	1774	0.6	MS, RI
57	Saussurea lactone	1799	1806	0.2	MS, RI
58	Hexadecanal	1813	1813	0.1	MS, RI
59	Neophytadiene	1832	1837	1.5	MS, RI
60	Hexahydrofarnesyl acetone	1839	1838	0.2	MS, RI
61	(*E*)-*N*-Isobutyldec-2-enamide	1850	1856	0.2	MS, RI
62	Diisobutyl phthalate	1868	1868	0.1	MS, RI
63	Isophytol	1942	1942	0.2	MS, RI
64	Cembrene	1948	1948	0.4	MS, RI
65	Neocembrene	1953	1951	0.4	MS, RI
66	*m*-Camphorene	1964	1960	1.1	MS, RI
67	α-Springene	1968	1969	0.3	MS, RI
68	Pentylcurcumene	1987	1992	0.5	MS, RI
69	*p*-Camphorene	1997	1995	0.6	MS, RI
70	Phyllocladene	2004	2011	0.1	MS, RI
71	Pimaradiene	2016	2019	0.2	MS, RI
72	Manool	2020	2027	0.2	MS, RI
73	Farnesyl butanoate	2027	2020	7.4	MS, RI
74	Thunbergol	2044	2047	0.2	MS, RI
75	Oleic Acid	2139	2140	0.3	MS, RI
76	Linoleic acid	2143	2144	0.4	MS, RI
77	Diisooctyl phthalate	2543	2545	0.1	MS, RI
	Monoterpene hydrocarbons			7.3	
	Oxygenated monoterpenes			3.0	
	Sesquiterpene hydrocarbons			45.2	
	Oxygenated sesquiterpenes			25.9	
	Diterpenes hydrocarbons			4.7	
	Oxygenated diterpenes			0.5	
	Total			98.0	

Notes: Compounds ^a^ are listed in order of their elution from a HP-5MS column; RI ^b^ (retention index): RI-non-isothermal Kovats retention indices on a HP-5MS column; RI ^c^ linear retention indices from the literature (NIST 14 Mass Spectra Library (Version 2.2 f) and WILEY’S Library of Mass spectra 9th Edition [18]) on a HP-5MS column; Identification ^d^: RI: Linear Retention index; MS: Mass Spectrometry.

**Table 2:** Antibacterial activity of GR-EO.

Microorganism	Diameter of the Inhibition Zones (mm) ^a^		MIC (mg/mL) ^b^		MBC (mg/mL) ^c^	
	GR-EO	CH	GR-EO	CH	GR-EO	CH
Gram positive						
*Bacillus subtilis* ATCC 6633	23.8 ± 0.7	29.6 ± 0.9	0.049	0.008	0.049	0.031
*Staphylococcus aureus* ATCC 6538	15.8 ± 0.5	27.1 ± 0.7	0.250	0.008	0.500	0.063
Gram negative						
*Escherichia coli* ATCC 25922	7.3 ± 0.8	31.5 ± 0.8	1.563	0.008	3.125	0.063
*Pseudomonas aeruginosa* ATCC 27853	24.1 ± 0.6	21.8 ± 0.5	0.049	0.063	0.049	0.063

The diameter of the inhibition zones (mm), including the disc diameter (6 mm), are given as the mean ± SD of triplicate experiments. Diameter of the inhibition zones ^a^ of GR-EO (1 mg/mL); positive control: CH: chloramphenicol (10 μg/disc); MIC ^b^: Minimal inhibitory concentration; MBC ^c^: Minimal bactericidal concentration.

**Table 3:** Fractional inhibitory concentrations indices (FICIs) of Chloramphenicol combined with GR-EO against tested bacterial strains.

Microorganism		MIC_a_ (μg/mL)	MIC_c_ (μg/mL)	FICI
*Bacillus subtilis* ATCC 6633	GR-EO	49.00	12.25	0.38 (S)
	CH	7.80	0.98	
*Staphylococcus aureus* ATCC 6538	GR-EO	250.00	62.50	1.50 (I)
	CH	7.80	9.75	
*Escherichia coli* ATCC 25922	GR-EO	1562.50	390.63	0.50 (S)
	CH	7.80	1.95	
*Pseudomonas aeruginosa* ATCC 27853	GR-EO	49.00	6.13	0.38 (S)
	CH	62.50	15.63	

MIC_a_: MIC of the sample alone or antibiotic alone; MIC_c_: MIC of the sample of the most effective combination; FICI: The fractional inhibitory concentration index; CH: Chloramphenicol. Abbreviations for interpretations: S, synergy; A, additivity; I, indifference.

**Table 4:** Fractional inhibitory concentrations indices (FICIs) of Streptomycin combined with GR-EO against tested bacterial strains.

Microorganism		MIC_a_ (μg/mL)	MIC_c_ (μg/mL)	FICI
*Bacillus subtilis* ATCC 6633	GR-EO	49.00	12.25	0.75 (A)
	SM	1.56	0.78	
*Staphylococcus aureus* ATCC 6538	GR-EO	250.00	31.28	0.38 (S)
	SM	62.50	15.63	
*Escherichia coli* ATCC 25922	GR-EO	1562.50	97.66	0.13 (S)
	SM	1000.00	62.50	
*Pseudomonas aeruginosa* ATCC 27853	GR-EO	49.00	6.13	0.38 (S)
	SM	6.25	1.56	

MIC_a_: MIC of the sample alone or antibiotic alone; MIC_c_: MIC of the sample of the most effective combination; FICI: The fractional inhibitory concentration index; SM: Streptomycin. Abbreviations for interpretations: S, synergy; A, additivity; I, indifference.

**Table 5:** Fractional inhibitory concentrations indices (FICIs) of Penicillin combined with GR-EO against tested bacterial strains.

Microorganism		MIC_a_ (μg/mL)	MIC_c_ (μg/mL)	FICI
*Escherichia coli* ATCC 25922	GR-EO	1562.50	390.63	0.50 (S)
	PNC	31.25	7.81	
*Pseudomonas aeruginosa* ATCC 27853	GR-EO	49.00	6.13	0.25 (S)
	PNC	2500.00	312.50	

MIC_a_: MIC of the sample alone or antibiotic alone; MIC_c_: MIC of the sample of the most effective combination; FICI: The fractional inhibitory concentration index; PNC: Penicillin. Abbreviations for interpretations: S, synergy; A, additivity; I, indifference.

**Table 6:** Fractional inhibitory concentrations indices (FICIs) of Ampicillin combined with GR-EO against tested bacterial strains.

Microorganism		MIC_a_ (μg/mL)	MIC_c_ (μg/mL)	FICI
*Escherichia coli* ATCC 25922	GR-EO	1562.50	781.25	1.00 (A)
	AM	12.50	6.25	
*Pseudomonas aeruginosa* ATCC 27853	GR-EO	49.00	6.13	0.38 (S)
	AM	1250.00	312.50	

MIC_a_: MIC of the sample alone or antibiotic alone; MIC_c_: MIC of the sample of the most effective combination; FICI: The fractional inhibitory concentration index; AM: Ampicillin. Abbreviations for interpretations: S, synergy; A, additivity; I, indifference.

## References

[1] Sheldon A.T. (2005). Antibiotic resistance: A survival strategy. Clin. Lab. Sci..

[2] Hemaiswarya S., Kruthiventi A.K., Doble M. (2008). Synergism between natural products and antibiotics against infectious diseases. Phytomedicine.

[3] Langeveld W.T., Veldhuizen E.J., Burt S.A. (2014). Synergy between essential oil components and antibiotics: A review. Crit. Rev. Microbiol..

[4] Miklasińska M., Kępa M., Wojtyczka R.D., Idzik D., Dziedzic A., Wąsik T.J. (2016). Catechin hydrate augments the antibacterial action of selected antibiotics against *Staphylococcus aureus* clinical strains. Molecules.

[5] Schäfer H., Wink M. (2009). Medicinally important secondary metabolites in recombinant microorganisms or plants: Progress in alkaloid biosynthesis. Biotechnol. J..

[6] Coutinho H.D.M., Costa J.G.M., Siqueira J.P., Lima E.O. (2008). In vitro anti-staphylococcal activity of *Hyptis martiusii* Benth against methicillin-resistant *Staphylococcus aureus*-MRSA strains. Rev. Bras. Farmacogn..

[7] Wang C.M., Chen H.T., Wu Z.Y., Jhan Y.L., Shyu C.L., Chou C.H. (2016). Antibacterial and synergistic activity of pentacyclic triterpenoids isolated from *Alstonia scholaris*. Molecules.

[8] Qin R., Xiao K., Li B., Jiang W., Peng W., Zheng J., Zhou H. (2013). The combination of catechin and epicatechin gallate from Fructus crataegi potentiates β-lactam antibiotics against methicillin-resistant *Staphylococcus aureus* (MRSA) in vitro and in vivo. Int. J. Mol. Sci..

[9] Taylor P.W., Stapleton P.D., Paul L.J. (2002). New ways to treat bacterial infections. Drug Discov. Today.

[10] Palaniappan K., Holley R.A. (2010). Use of natural antimicrobials to increase antibiotic susceptibility of drug resistant bacteria. Int. J. Food Microbiol..

[11] Moussaoui F., Alaoui T. (2016). Evaluation of antibacterial activity and synergistic effect between antibiotic and the essential oils of some medicinal plants. Asian Pac. J. Trop. Biomed..

[12] Bassole I.H.N., Juliani H.R. (2012). Essential oils in combination and their antimicrobial properties. Molecules.

[13] Maruzzella J.C., Bloch A. (1959). The effect of antibiotic-essential oil combinations on *Staphylococcus aureus*. Naturwissenschaften.

[14] Santiesteban-LóPez A., Palou E., LóPez-Malo A. (2007). Susceptibility of food-borne bacteria to binary combinations of antimicrobials at selected *a*_w_ and pH. J. Appl. Microbiol..

[15] Stefanakis M.K., Touloupakis E., Anastasopoulos E., Ghanotakis D., Katerinopoulos H.E., Makridis P. (2013). Antibacterial activity of essential oils from plants of the genus Origanum. Food Control.

[16] Davis A.P., Govaerts R., Bridson D.M., Ruhsam M., Moat J., Brummitt N.A. (2009). A global assessment of distribution, diversity, endemism, and taxonomic effort in the Rubiaceae. Ann. Mo. Bot. Gard..

[17] Portillo A., Vila R., Freixa B., Adzet T., Canigueral S. (2001). Antifungal activity of Paraguayan plants used in traditional medicine. J. Ethnopharmacol..

[18] Hammami S., Jmii H., Mokni R.E., Khmiri A., Faidi K., Dhaouadi H., Aouni M.H., Aouni M., Joshi R.K. (2015). Essential oil composition, antioxidant, cytotoxic and antiviral activities of *Teucrium pseudochamaepitys* Growing Spontaneously in Tunisia. Molecules.

[19] Odds F.C. (2003). Synergy, antagonism, and what the chequerboard puts between them. J. Antimicrob. Chemother..

[20] Dahham S.S., Tabana Y.M., Iqbal M.A., Ahamed M.B.K., Ezzat M.O., Majid A.S.A., Majid A.M.S.A. (2015). The anticancer, antioxidant and antimicrobial properties of the sesquiterpene beta-caryophyllene from the essential oil of *Aquilaria crassna*. Molecules.

[21] Xiong L., Peng C., Zhou Q.M., Wan F., Xie X.F., Guo L., Li X.H., He C.J., Dai O. (2013). Chemical composition and antibacterial activity of essential oils from different parts of *Leonurus japonicus* Houtt. Molecules.

[22] Li X., Wang G., Zhao J., Ding H., Cunningham C., Chen F., Flynn D., Reed E., Li Q. (2005). Antiproliferative effect of β-elemene in chemoresistant ovarian carcinoma cells is mediated through arrest of the cell cycle at the G2-M phase. Cell. Mol. Life Sci..

[23] Wang G., Li X., Huang F., Zhao J., Ding H., Cunningham C., Coad J.E., Flynn D.C., Reed E., Li Q.Q. (2005). Antitumor effect of beta-elemene in non-small-cell lung cancer cells is mediated via induction of cell cycle arrest and apoptotic cell death. Cell. Mol. Life Sci..

[24] Wang Y., Deng Y., Mao S., Jin S., Wang J., Bi D. (2005). Characterization and body distribution of beta-elemene solid lipid nanoparticles (SLN). Drug Dev. Ind. Pharm..

[25] Shakeri A., Khakdan F., Soheili V., Sahebkar A., Shaddel R., Asili J. (2016). Volatile composition, antimicrobial, cytotoxic and antioxidant evaluation of the essential oil from *Nepeta sintenisii* Bornm. Ind. Crops Prod..

[26] Burt S. (2004). Essential oils: Their antibacterial properties and potential applications in foods—A review. Int. J. Food Microbiol..

[27] Delaquis P.J., Stanich K., Girard B., Mazza G. (2002). Antimicrobial activity of individual and mixed fractions of dill, cilantro, coriander and eucalyptus essential oils. Int. J. Food Microbiol..

[28] Davidson P.M., Parish M.E. (1989). Methods for testing the efficacy of antimicrobials. Food Technol.-Chicago.

[29] Gill A.O., Delaquis P., Russo P., Holley R.A. (2002). Evaluation of antilisterial action of cilantro oil on vacuum packed ham. Int. J. Food Microbiol..

[30] Mourey A., Canillac N. (2002). Anti-listeria monocytogenes activity of essential oils components of conifers. Food Control.

[31] Ji Y., Yoon J.W., Hovde C.J. (2010). A brief overview of *Escherichia coli* O157:H7 and its plasmid O157. J. Microbiol. Biotechnol..

[32] Rossolini G.M., Mantengoli E. (2005). Treatment and control of severe infections caused by multiresistant *Pseudomonas aeruginosa*. Clin. Microbiol Infect..

[33] Lambert P.A. (2002). Mechanisms of antibiotic resistance in *Pseudomonas aeruginosa*. J. R. Soc. Med..

[34] Tong S.Y., Davis J.S., Eichenberger E., Holland T.L., Fowler F.V. (2015). *Staphylococcus aureus* infections: Epidemiology, pathophysiology, clinical manifestations, and management. Clin. Microbiol. Rev..

[35] Marshall N.E., Ziegler H.K. (1989). Role of lipopolysaccharide in induction of Ia expression during infection with gram-negative bacteria. Infect. Immun..

[36] Marletta M.A. (1993). Nitric oxide synthase structure and mechanism. J. Biol. Chem..

[37] Tung Y.T., Chua M.T., Wang S.Y., Chang S.T. (2008). Anti-inflammation activities of essential oil and its constituents from indigenous cinnamon (*Cinnamomum osmophloeum*) twigs. Bioresour. Technol..

[38] Ku C.M., Lin J.Y. (2013). Anti-inflammatory effects of 27 selected terpenoid compounds tested through modulating Th1/Th2 cytokine secretion profiles using murine primary splenocytes. Food Chem..

[39] Gertsch J., Leonti M., Raduner S., Racz I., Chen J.Z., Xie X.Q., Altmann K.H., Karsak M., Zimmer A. (2008). Beta-caryophyllene is a dietary cannabinoid. Proc. Natl. Acad. Sci. USA.

[40] Clinical & Laboratory Standards Institute (2012). Performance standards for antibacterial disk susceptibility tests. Approved Standard.

[41] Eloff J.N. (1998). A sensitive and quick microplate method to determine the minimal inhibitory concentration of plant extracts for bacteria. Planta Med..

[42] Andrews J.M. (2001). Determination of minimum inhibitory concentrations. J. Antimicrob. Chemother..

[43] White R.L., Burgess D.S., Manduru M., Bosso J.A. (1996). Comparison of three different in vitro methods of detecting synergy: Time-kill, checkerboard, and E test. Antimicrob. Agents Chemother..

[44] Green L.C., Wagner D.A., Glogowski J., Skipper P.L., Wishnok J.S., Tannenbaum S.R. (1982). Analysis of nitrate, nitrite, and [15N]nitrate in biological fluids. Anal. Biochem..

